# Chromatic Stability of Three Photopolymerizable Resins for Additive Manufacturing Following Immersion in Artificial Saliva: A Comparative Spectrophotometric Analysis

**DOI:** 10.3390/jfb17080386

**Published:** 2026-08-05

**Authors:** Adina-Bianca Bulai, Alexia-Ecaterina Cârstea, Lucian-Toma Ciocan, Andreia Cucuruz, Nicoleta Badea, Vlad-Gabriel Vasilescu, Alexandra Ripszky, Ana-Maria Cristina Țâncu, Silviu-Mirel Pițuru, Marina Imre

**Affiliations:** 1 Faculty of Medical Engineering, National University of Science and Technology Politehnica Bucharest, 1-7 Gh. Polizu Street, District 1, 011061 Bucharest, Romania; adina_bianca.bulai@stud.fim.upb.ro; 2 Discipline of Dental Prosthesis Technology, Faculty of Dentistry, “Carol Davila” University of Medicine and Pharmacy, Dionisie Lupu Street, No. 37, District 2, 020021 Bucharest, Romania; alexia-ecaterina.carstea2023@stud.umfcd.ro (A.-E.C.); vlad.vasilescu@umfcd.ro (V.-G.V.); 3 Faculty of Chemical Engineering and Biotechnologies, National University of Science and Technology Politehnica Bucharest, Polizu No 1, 011061 Bucharest, Romania; nicoleta.badea@gmail.com; 4 Research Centre for Environmental Protection and Ecofriendly Technologies (CPMTE), National University of Science and Technology Politehnica Bucharest, Polizu No 1, 011061 Bucharest, Romania; 5 Department of Biochemistry, Faculty of Dental Medicine, University of Medicine and Pharmacy Carol Davila, 37 Dionisie Lupu Street, District 2, 020021 Bucharest, Romania; alexandra.ripszky@umfcd.ro; 6 Department of Prosthodontics, Faculty of Dentistry, “Carol Davila” University of Medicine and Pharmacy, 37 Dionisie Lupu Street, District 2, 020021 Bucharest, Romania; anamaria.tancu@umfcd.ro (A.-M.C.Ț.); marina.imre@umfcd.ro (M.I.); 7 Department of Professional Organization and Medical Legislation-Malpractice, “Carol Davila” University of Medicine and Pharmacy, 37 Dionisie Lupu Street, District 2, 020021 Bucharest, Romania; silviu.pituru@umfcd.ro

**Keywords:** color stability, 3D printing, dental resin, CIELAB, artificial saliva, spectrophotometry, provisional restoration

## Abstract

Objective: This study compared the color stability of three photopolymerizable resins (Anycubic Bio Resin White, V-Print c&b temp, GC Temp Print) after immersion in artificial saliva at 37 °C. Materials and Methods: Thirty-six disc specimens (10 mm × 1.5 mm) were printed on a Planmeca Creo C5 LCD printer and divided into control (T0), 24 h (T24), and 72 h (T72) groups (*n* = 4). CIELAB coordinates were measured spectrophotometrically, and ΔE was calculated using the CIE76 formula. Results: Material was the dominant factor for all color parameters (η^2^*p* = 0.774–0.964, all *p* < 0.001), while immersion time was not significant (all *p* > 0.05). Mean ΔE at T24/T72 was 0.90/0.66 (Anycubic), 2.25/3.79 (VOCO), and 4.48/3.58 (GC). The Kruskal–Wallis test detected an overall difference among materials at T72 (H(2) = 7.423, *p* = 0.024); however, no pairwise comparison remained significant after Bonferroni correction. At T24, one of four Anycubic specimens exceeded the perceptibility threshold (ΔE = 1.2), whereas 75% of GC Temp Print specimens exceeded the clinical acceptability threshold (ΔE > 3.7). Conclusions: Material composition was the primary determinant of chromatic behavior; resin selection remains critical for the aesthetic performance of 3D-printed provisional restorations.

## 1. Introduction

The adoption of additive manufacturing technologies in prosthodontic dentistry has expanded the range of photopolymerizable resins available for the fabrication of provisional restorations [1]. Layer-by-layer polymerization using digital light processing (DLP) or liquid crystal display (LCD) systems enables the production of geometrically precise prosthetic devices with reduced material waste and shorter turnaround times [2,3]. The clinical longevity of such restorations depends not only on dimensional accuracy but also on the ability of the polymer matrix to resist degradation under the physicochemical conditions of the oral environment [4,5]. The oral cavity is a thermodynamically complex environment, marked by temperature shifts, pH changes, enzymatic activity, and constant saliva exposure [6,7,8,9]. These factors act synergistically to promote hydrolytic degradation of the polymer network through water sorption and plasticization of the organic matrix [10,11]. In the context of 3D-printed resins, the stratified architecture inherent to additive fabrication may introduce micro-voids between successive layers, providing additional pathways for fluid ingress [9,12]. Water molecules that penetrate the polymer network can disrupt intermolecular forces and facilitate the leaching of residual monomers, all of which may compromise both the structural integrity and the optical properties of the restoration [10,13,14]. Beyond its water content, saliva is a compositionally complex biological fluid whose organic and inorganic constituents actively participate in this degradation. Salivary proteins, mucins, and glycoproteins adsorb onto the polymer surface to form an acquired pellicle that modifies surface wettability and light scattering, whereas salivary esterases and other hydrolytic enzymes can cleave ester linkages within methacrylate networks, accelerating residual-monomer release and surface softening. In parallel, salivary electrolytes and ions (Na^+^, K^+^, Ca^2+^, Cl^−^ and phosphate) govern the ionic strength and buffering capacity of the medium and thereby modulate water sorption, solubility, and the leaching equilibrium of the matrix. Collectively, these constituents can influence the optical behaviour of a restoration to a greater extent than water alone [15].

Chromatic stability serves as an important clinical marker of material performance, since noticeable color changes can indicate underlying structural changes in the polymer matrix and may directly influence patient satisfaction with provisional restorations [5,16]. Unlike extrinsic staining caused by dietary pigments such as coffee or tea, prolonged contact with salivary fluids can induce intrinsic modifications that are more subtle, including opacity shifts and lightness reduction, which reflect the penetration of water molecules into the polymer network [5,10,17]. In vivo, provisional restorations are additionally colonized by oral biofilms; chromogenic bacteria embedded within these biofilms can deposit pigmented by-products on the restoration surface, further compromising color stability and translucency [18]. Although biofilm-mediated staining was deliberately excluded from the present protein-free static model, acknowledging this factor is important both to justify the experimental design and to delimit the clinical extrapolation of its findings [7,14].

Building on early perceptual frameworks such as the Munsell color system, which described color through hue, value, and chroma but depended on subjective visual matching and offered limited instrumental reproducibility [19], the CIELAB color space, defined by the Commission Internationale de l’Eclairage in 1976, was developed as a device-independent, mathematically defined system that maps Munsell’s perceptual dimensions onto quantitative coordinates: L* (corresponding to Munsell value), while a* and b* encode the chromatic information analogous to hue and chroma [20,21]. In this system, color is described by three coordinates: L* (lightness, ranging from 0 for black to 100 for white), a* (the red-green axis), and b* (the yellow-blue axis). Total color change is expressed as ΔE, calculated using the CIE76 formula: ΔE = √(ΔL*^2^ + Δa*^2^ + Δb*^2^) [21,22,23,24].

Two clinically relevant thresholds have been established for interpreting ΔE values in the context of dental materials. The perceptibility threshold (ΔE ≈ 1.2) represents the smallest color difference detectable by the human eye, while the acceptability threshold (ΔE ≈ 3.7) defines the upper limit beyond which a restoration is considered aesthetically unacceptable and warrants replacement [2,25]. The molecular composition of the resin matrix plays a central role in determining susceptibility to hydrolytic degradation. Methacrylate-based systems containing urethane dimethacrylate (UDMA) as the primary monomer tend to form dense, highly crosslinked networks with limited water uptake [26]. In contrast, resins with elevated concentrations of hydrophilic diluent monomers such as triethylene glycol dimethacrylate (TEGDMA) or triethylene glycol diacrylate exhibit greater fluid absorption, which may facilitate both intrinsic discoloration and mechanical softening over time [4,27,28]. The type and concentration of photoinitiator also influence chromatic behavior; modern phosphine oxide-based systems activated at 405 nm eliminate the need for tertiary amines, thereby reducing the yellowing tendency associated with amine oxidation [29]. Despite the growing body of literature on the mechanical and biological properties of 3D-printed dental resins, comparative data on the chromatic response of different resin categories to prolonged salivary exposure remain limited [30,31,32,33]. Most existing studies focus on extrinsic staining agents (coffee, tea, wine) [34,35,36,37] or employ thermocycling protocols, while fewer investigations address the baseline hydrolytic color shift induced by artificial saliva alone [5,16,38,39,40]. Recent investigations have begun to characterize the translucency, gloss and long-term optical behaviour of additively manufactured provisional resins, showing that specimen thickness, printing orientation, post-curing regimen, and artificial aging substantially modulate translucency and post-immersion color shifts [41,42]; however, these studies predominantly rely on thermocycling or acidic and tooth-brushing challenges [43], so that the isolated contribution of salivary immersion to intrinsic color change remains comparatively underexplored. By deliberately isolating the hydrolytic effect of artificial saliva, without the confounding influence of dietary chromogens or thermocycling, the present study establishes a baseline reference for the intrinsic chromatic behaviour of these resin categories, against which the additional impact of extrinsic and thermal stressors can subsequently be separated and quantified. This isolation of a single degradation pathway, together with the head-to-head comparison of three distinct printable resin categories under identical conditions, constitutes the principal novelty of the study.

The three resins examined here were selected to represent three clinically distinct categories of printable material. Biocompatible skin-contact acrylate resins, although not primarily marketed for intraoral prosthetics, are increasingly repurposed for provisional applications owing to their low cost and reported biocompatibility [44]. Urethane-dimethacrylate-based provisional resins such as V-Print c&b temp are certified medical devices formulated for long-term temporaries and are generally reported to combine acceptable esthetics with favourable mechanical and color stability [45]. High-UDMA nanocomposite printable materials such as GC Temp Print are designed to couple a densely crosslinked matrix with filler reinforcement, although their optical stability has been shown to be sensitive to printing orientation and post-curing protocol [42,46]. A direct comparison of these three categories under identical salivary-immersion conditions has rarely been reported, which further motivates the present investigation.

The aim of the present study was to evaluate and compare the chromatic stability of three commercially available photopolymerizable resins representing distinct compositional categories (a biocompatible acrylate resin designed for skin-contact applications, a premium urethane dimethacrylate-based provisional resin, and a nanocomposite UDMA-based printable composite) following static immersion in artificial saliva at 37 °C for 24 and 72 h. Stability was assessed through spectrophotometric analysis of CIELAB parameters (L*, a*, b*) and calculation of total color change (ΔE) relative to baseline values. The null hypothesis was that there would be no statistically significant difference in ΔE among the three materials at either immersion time point.

## 2. Materials and Methods

### 2.1. Study Design

**Figure 1:**
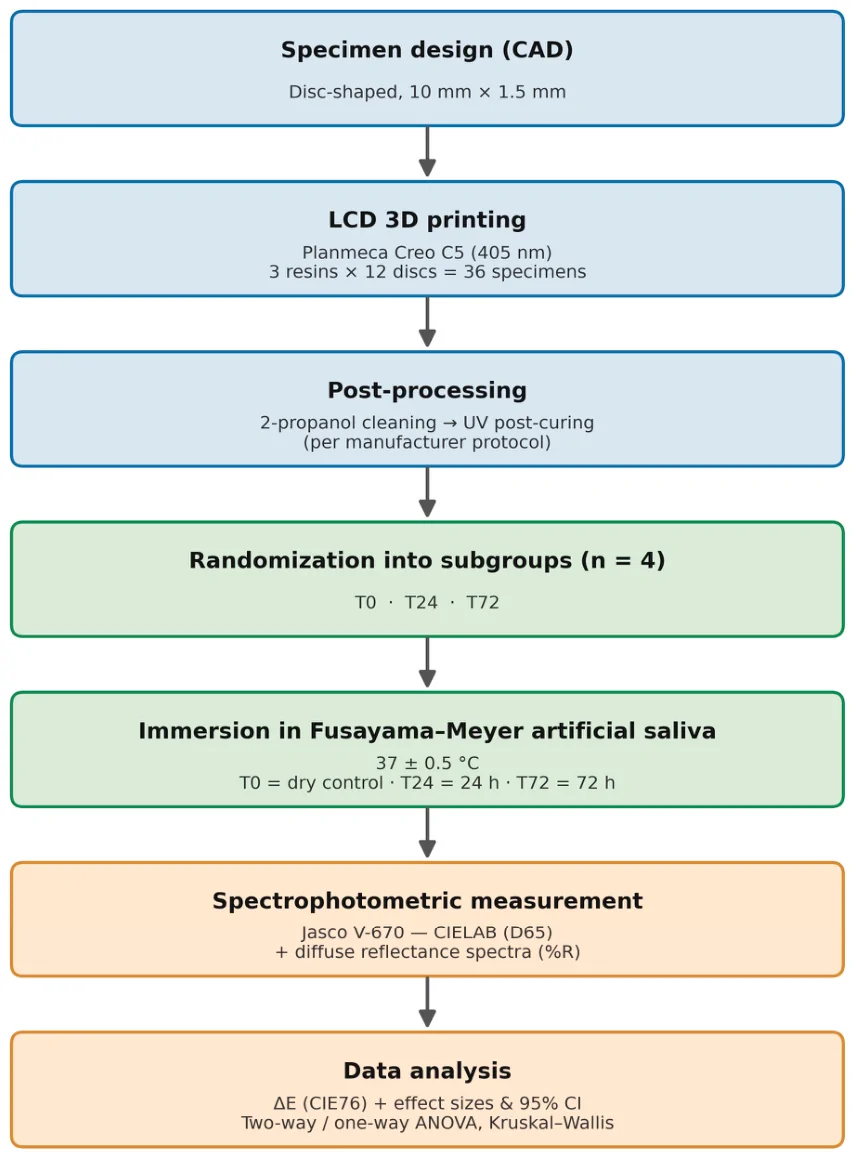
Schematic representation of the experimental workflow of the study, from specimen design and additive manufacturing through standardized post-processing, randomization and immersion in artificial saliva to spectrophotometric colour measurement and statistical analysis.

This in vitro experimental study was designed using a factorial between-subjects design with two independent variables: Material (three levels: Anycubic Bio Resin White, V-Print c&b temp, GC Temp Print) and Immersion Time (three levels: T0, T24, T72). The primary outcome variables were the CIELAB chromatic coordinates (L*, a*, b*) and total color change (ΔE). Specimens were randomly assigned to subgroups with four specimens per cell (*n* = 4), yielding a total of 36 disc-shaped specimens. An overview of the complete experimental workflow, from specimen design and additive manufacturing through post-processing, grouping and immersion in artificial saliva to spectrophotometric measurement and statistical analysis, is provided in Figure 1.

### 2.2. Materials

Three photopolymerizable resins were selected to represent distinct compositional profiles and intended clinical applications (Table 1).

The three materials represent, respectively, a skin-contact biocompatible acrylate resin (Anycubic Bio Resin White), a Class IIa urethane-dimethacrylate provisional medical device (V-Print c&b temp), and a high-UDMA nanocomposite printable material (GC Temp Print). Their manufacturer, intended use, monomer and photoinitiator chemistry, and key physical properties are summarized in Table 1; compositional data were obtained from the respective manufacturers’ technical and safety data sheets [44,45,46], and the specific monomer identities and weight fractions of the Anycubic resin are not disclosed by the manufacturer [44].

The immersion medium was Fusayama–Meyer artificial saliva, prepared from analytical-grade reagents (Sigma-Aldrich; Merck KGaA, Darmstadt, Germany); its composition is given in Table 2. The rationale for selecting this protein-free formulation, which reproduces the ionic strength and buffering capacity of natural saliva more closely than distilled water, is presented in the Introduction and Discussion [47].

### 2.3. Specimen Fabrication

Disc-shaped specimens (10 mm diameter, 1.5 mm thickness) were designed using the open-source 3D modelling software Blender (v.3.3 LTS; Blender Foundation, Amsterdam, The Netherlands) and fabricated on a Planmeca Creo C5 medical-grade LCD 3D printer (Planmeca Oy, Helsinki, Finland) operating at a wavelength of 405 nm. A total of 12 discs were printed for each material. Following printing, all specimens were removed from the build platform and subjected to a standardized post-processing protocol. First, each disc was immersed in 2-propanol (>90% purity) to remove uncured surface resin. Second, specimens were subjected to UV post-curing to increase monomer conversion and lower residual porosity. All materials were processed according to the respective manufacturer’s instructions for post-curing time and UV intensity. Solvent immersion in 2-propanol was preferred over mechanical abrasion for the removal of uncured surface resin because it follows the manufacturer’s recommended post-processing workflow and cleans the whole surface uniformly without altering specimen thickness, surface topography, or gloss; variables would otherwise confound the subsequent colorimetric and reflectance measurements.

### 2.4. Grouping and Immersion Protocol

The 12 specimens of each material were randomly allocated into three subgroups of four (*n* = 4). The first subgroup (T0) was the unimmersed control and was stored under dry, ambient conditions. The second subgroup (T24) was immersed in artificial saliva for 24 h, and the third subgroup (T72) was immersed for 72 h. All immersed specimens were placed in aluminum trays to prevent overlapping and ensure complete surface exposure to the medium. The trays were maintained in a Nahita Model 601/5 digital water bath (Auxilab S.L., Beriain, Spain) set at 37.0 ± 0.5 °C throughout the immersion period.

### 2.5. Chromatic Analysis

Color measurements were performed using a calibrated Jasco V-670 UV-VIS-NIR spectrophotometer (JASCO Corporation, Tokyo, Japan) operating in the CIELAB color space with D65 standard illuminant. Prior to each measurement session, the instrument was calibrated against the manufacturer-supplied white reference tile. For each specimen, three readings were taken at different points on the disc surface, and the mean values of L*, a*, and b* were recorded. The T0, T24, and T72 groups consisted of independent specimens. Therefore, total color difference (ΔE) for each specimen at T24 and T72 was calculated relative to the mean baseline CIELAB values (T0) of the corresponding material group rather than from repeated measurements of the same specimens, using the CIE76 formula:

ΔE = √[(ΔL*)^2^ + (Δa*)^2^ + (Δb*)^2^]

where ΔL* = L*specimen-mean L*T0, and analogously for Δa* and Δb*. Individual ΔE values were calculated for each specimen relative to the mean baseline value of the corresponding T0 group. Because T0, T24, and T72 consisted of independent specimens, these ΔE values represent exploratory between-group estimates rather than true within-specimen color changes. The obtained ΔE values were interpreted against the established perceptibility threshold (ΔE = 1.2) and clinical acceptability threshold (ΔE = 3.7) [25].

### 2.6. Statistical Analysis

Statistical analyses were performed using IBM SPSS Statistics (version 29; IBM Corp., Armonk, NY, USA). The significance level was set at α = 0.05 for all tests. Descriptive statistics (mean, standard deviation, median, minimum, maximum) were calculated for each variable within each Material × Time cell. The normality of distributions within each cell was assessed using the Shapiro–Wilk test, which is recommended for small sample sizes. Homogeneity of variances was evaluated with the Levene test. A two-way between-subjects analysis of variance (ANOVA) was conducted to examine the main effects of Material and Immersion Time and their interaction on each CIELAB parameter (L*, a*, b*) separately. Where the ANOVA yielded a significant result, pairwise comparisons were performed using Bonferroni-adjusted tests. Partial eta-squared (η^2^p) was reported as a measure of effect size, interpreted as small (<0.06), medium (0.06–0.14), or large (>0.14). For ΔE, which is defined only at T24 and T72, one-way ANOVA with Material as the grouping factor was performed separately at each time point. Where variances were heterogeneous (Levene *p* < 0.05), the Welch robust test and Games-Howell post hoc comparisons were employed. Cohen’s d was calculated for pairwise comparisons to quantify practical differences between materials. Ninety-five percent confidence intervals (95% CI) were additionally calculated for the mean ΔE of each material at each immersion time point. Given the small sample size (*n* = 4 per cell), which limits the statistical power of parametric tests, non-parametric Kruskal–Wallis tests were additionally performed on ΔE at each time point to verify the robustness of the parametric findings. Where Kruskal–Wallis yielded significant results, pairwise Mann–Whitney U tests with Bonferroni-adjusted significance levels (α/3 = 0.0167) were conducted. Finally, a descriptive analysis of clinical relevance was performed by determining the proportion of specimens in each group exceeding the perceptibility (ΔE > 1.2) and acceptability (ΔE > 3.7) thresholds. Because departures from normality were limited to two of the nine b* cells and were driven by individual specimens, and because the design used equal group sizes (a condition under which ANOVA remains comparatively robust), parametric models were retained as the primary analysis. The Kruskal–Wallis test was applied to ΔE not because these data were non-normal, but as a pre-planned, distribution-free cross-check of the parametric result, warranted by the low statistical power associated with *n* = 4 per cell. Effect sizes (η^2^p, η^2^ and Cohen’s d) are reported alongside all *p*-values, and their interpretation, together with the comparison between the parametric and non-parametric outcomes, is presented in the Discussion rather than the Results. A sensitivity analysis (one-way ANOVA, three groups, α = 0.05) further contextualized the sample size: even for the very large effects observed for ΔE (Cohen’s f ≈ 0.9), the power achieved with four specimens per group was only approximately 0.65, whereas detecting a conventionally large effect (Cohen’s f = 0.40) with α = 0.05 and 80% power would require approximately 22 specimens per group (66 specimens in total). Likewise, detecting a medium effect (f = 0.25) would require approximately 53 specimens per group (159 specimens in total). These estimates confirm the exploratory, screening nature of the present design and are intended to guide the sample size of future confirmatory studies.

## 3. Results

### 3.1. Descriptive Statistics

The descriptive statistics for CIELAB parameters across all material and time combinations are presented in Table 3. Baseline (T0) values revealed substantial differences in inherent optical properties among the three resins. VOCO specimens exhibited the lowest lightness (L* = 88.49 ± 0.92) and the highest yellowness (b* = 15.52 ± 1.88), while Anycubic specimens were the lightest (L* = 94.32 ± 0.42) with a moderate yellowness (b* = 6.12 ± 0.41). GC Temp Print specimens showed intermediate lightness (L* = 91.67 ± 1.96) but also the highest within-group variability in baseline values, with L* ranging from 90.24 to 94.48. On the chromatic axes, Anycubic exhibited negative a* values (a* = −1.85 ± 0.42), indicating a greenish tint; VOCO showed positive a* values (a* = 2.14 ± 0.29), reflecting a slight reddish component; and GC was near-neutral on this axis (a* = 0.47 ± 0.21).

### 3.2. Distributional Assumptions

Shapiro–Wilk tests did not detect significant departures from normality within each cell for L* (all W ≥ 0.836, all *p* > 0.05) or a* (all W ≥ 0.926, all *p* > 0.05). For b*, two cells (Anycubic T0 and Anycubic T24) showed marginally significant departures from normality (W = 0.751 and 0.750, *p* = 0.039), which appeared to be driven by an individual specimen with a more extreme value in each cell; because no formal outlier test was applied, these departures are interpreted with caution. ΔE distributions were likewise compatible with normality (all *p* > 0.14), and Levene tests did not indicate heterogeneity of variances for the three CIELAB parameters across the nine-cell design (all *p* > 0.29). Given the small subgroup size (*n* = 4), these tests are underpowered; accordingly, non-significant results are regarded as an absence of strong evidence against normality and homogeneity rather than as positive confirmation of these assumptions. The retention of parametric analyses, supplemented by the non-parametric Kruskal–Wallis cross-check, is justified in the Statistical Analysis and interpreted in the Discussion.

### 3.3. Two-Way ANOVA (Material × Immersion Time)

The two-way between-subjects ANOVA revealed a highly significant main effect of Material on all three CIELAB parameters (Table 4). For L*, the main effect of Material was significant, F(2, 27) = 46.237, *p* < 0.001, η^2^p = 0.774. The effect was even more pronounced for a*, F(2, 27) = 365.796, *p* < 0.001, η^2^p = 0.964, and for b*, F(2, 27) = 55.905, *p* < 0.001, η^2^p = 0.805. In contrast, the main effect of Immersion Time was not statistically significant for any parameter: L* (F(2, 27) = 1.563, *p* = 0.228, η^2^p = 0.104), a* (F(2, 27) = 0.519, *p* = 0.601, η^2^p = 0.037), and b* (F(2, 27) = 2.664, *p* = 0.088, η^2^p = 0.165). The Material × Time interaction was not statistically significant for any parameter (all *p* > 0.18); although the partial eta-squared for b* was in the large range (η^2^p = 0.197), the interaction did not reach significance and is therefore reported here as a non-significant effect.

The Bonferroni-corrected pairwise comparisons for the Material main effect on L* showed that Anycubic significantly differed from VOCO (*p* < 0.001), and VOCO differed from GC (*p* < 0.001). However, the comparison between Anycubic and GC was not significant after correction (*p* = 0.142). For a*, all three pairwise comparisons were significant (all *p* < 0.001). For b*, Anycubic differed from VOCO (*p* < 0.001), and VOCO differed from GC (*p* < 0.001), while Anycubic and GC did not differ significantly (*p* = 0.921).

### 3.4. Analysis of Total Color Change (ΔE)

At T24, the one-way ANOVA on ΔE did not reach statistical significance, F(2, 9) = 2.820, *p* = 0.112, although the effect size was large (η^2^ = 0.385). The mean ΔE values were 0.90 ± 0.54 for Anycubic, 2.25 ± 2.04 for VOCO, and 4.48 ± 3.07 for GC Temp Print (95% CI: 0.04–1.76 for Anycubic, −1.00–5.50 for VOCO, and −0.40–9.36 for GC). Despite the magnitude of the group differences, the high within-group variability (particularly in the GC group, where ΔE ranged from 0.55 to 7.99) precluded detection of a statistically significant effect with *n* = 4. Pairwise comparisons revealed large Cohen’s d values for Anycubic versus VOCO (d = 0.91) and Anycubic versus GC (d = 1.62), both exceeding the conventional threshold for a large effect (d > 0.80), although none reached the Bonferroni-corrected significance level. At T72, the one-way ANOVA approached significance, F(2, 9) = 3.631, *p* = 0.070, with a large effect size (η^2^ = 0.447). Mean ΔE values were 0.66 ± 0.49 (Anycubic), 3.79 ± 2.49 (VOCO), and 3.58 ± 1.91 (GC) (95% CI: −0.12–1.44, −0.17–7.75, and 0.54–6.62, respectively). The Cohen’s d for Anycubic versus VOCO was 1.74, and for Anycubic versus GC was 2.09, both representing very large effects. VOCO and GC did not differ from each other (d = 0.09). Non-parametric Kruskal–Wallis tests corroborated the T24 finding (H(2) = 2.462, *p* = 0.292) but reached significance at T72 (H(2) = 7.423, *p* = 0.024). Pairwise Mann–Whitney U comparisons were examined to localize this difference; however, with only four specimens per group, the smallest attainable two-tailed *p*-value (≈0.029) exceeds the Bonferroni-adjusted threshold (α = 0.0167), so no pairwise comparison could reach formal significance. Descriptively, the mean-rank pattern (Anycubic = 2.50; VOCO = 8.75; GC = 8.25) indicates that the overall effect was driven by the separation of Anycubic from both dental resins, with VOCO and GC occupying comparable upper ranks. As the three time-point groups comprised independent specimens, these ΔE values are exploratory between-group estimates rather than within-specimen colour changes and are interpreted accordingly.

### 3.5. Clinical Analysis

The proportion of specimens exceeding established clinical thresholds is summarized in Table 5. All Anycubic specimens remained below the perceptibility threshold (ΔE < 1.2) at T72, and only one specimen (25%) exceeded it at T24. In contrast, three of four GC specimens (75%) exceeded the clinical acceptability threshold (ΔE > 3.7) at T24, and two of four (50%) did so at T72. For VOCO, the proportion exceeding the perceptibility threshold increased from 75% at T24 to 100% at T72, while one specimen (25%) exceeded the acceptability threshold at each time point. Because each subgroup comprised only four specimens, these proportions are presented descriptively: a change in a single specimen alters the percentage by 25%, and the values should therefore be interpreted with corresponding caution, as addressed in the Limitations.

### 3.6. Diffuse Reflectance Spectral Analysis

**Figure 2:**
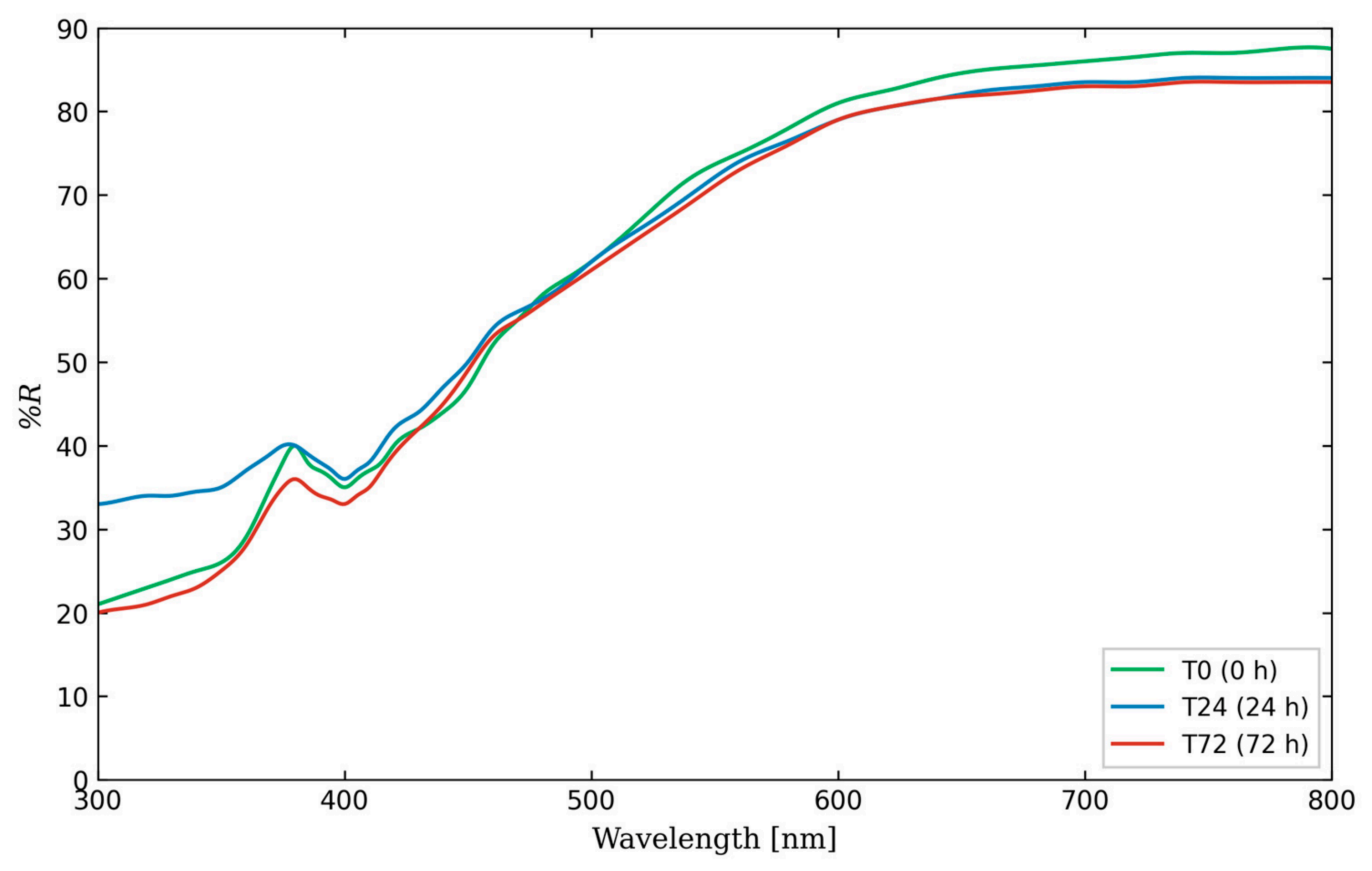
Diffuse reflectance spectra (%R) of VOCO V-Print c&b temp specimens at baseline (T0, green), after 24 h immersion (T24, blue), and after 72 h immersion (T72, red) in artificial saliva at 37 °C. Wavelength range: 300–800 nm.

To complement the colorimetric assessment, diffuse reflectance spectra (%R) were acquired across the 300–800 nm range using a Jasco V-670 UV-VIS-NIR spectrophotometer (JASCO Corporation, Tokyo, Japan) equipped with an integrating sphere. For each material, the spectra of a single representative specimen per group at T0, T24, and T72 were superimposed to visualize immersion-induced changes across the full visible and near-UV spectrum. The reflectance spectrum of VOCO V-Print c&b temp (Figure 2) exhibited a linear optical transition, with reflectance increasing progressively from approximately 20–33% at 300 nm to a plateau of 83–87% in the 650–800 nm region. A characteristic shoulder was observed near 380 nm, followed by a slight depression at approximately 400 nm, consistent with residual photoinitiator absorption in the near-UV range. The three time-point traces converged closely across the entire spectral range, with the T0 specimen reaching the highest plateau reflectance (~87%) and the T24 and T72 specimens stabilizing at approximately 83–84% (Figure 2). Because only one representative specimen per group was analysed rather than the full set, the reflectance spectra are presented as qualitative, supplementary observations that complement, but do not replace, the quantitative CIELAB analysis; their generalizability is correspondingly limited by this sampling.

**Figure 3:**
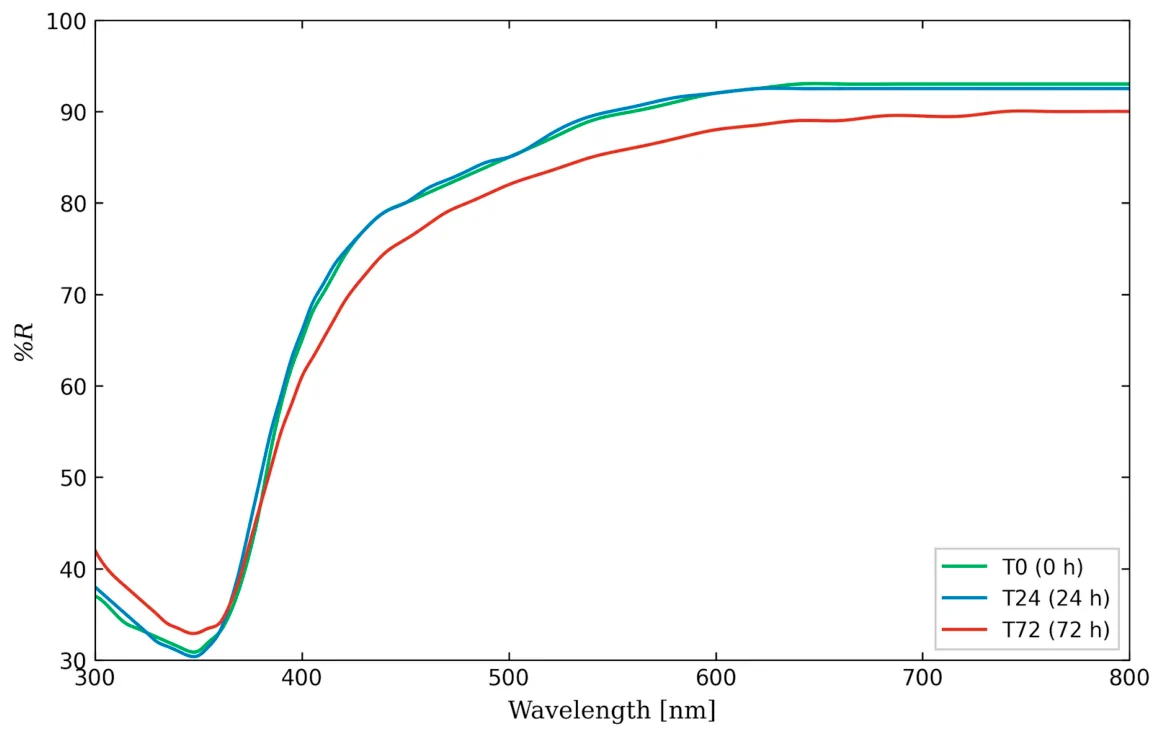
Diffuse reflectance spectra (%R) of GC Temp Print specimens at baseline (T0, green), after 24 h immersion (T24, blue), and after 72 h immersion (T72, red) in artificial saliva at 37 °C. Wavelength range: 300–800 nm.

In contrast, the reflectance spectrum of GC Temp Print (Figure 3) revealed a more dynamic post-immersion response. The UV-A domain (300–400 nm) featured an absorption band centered at approximately 350 nm, producing a reflectance minimum of 30–33%. At 24 h of immersion, the spectral profile remained largely superimposable on the baseline. However, at 72 h, the spectrum exhibited a pronounced downward shift across the entire visible range (400–800 nm), with the plateau reflectance decreasing from approximately 93% (T0) to 90% (T72) (Figure 3).

**Figure 4:**
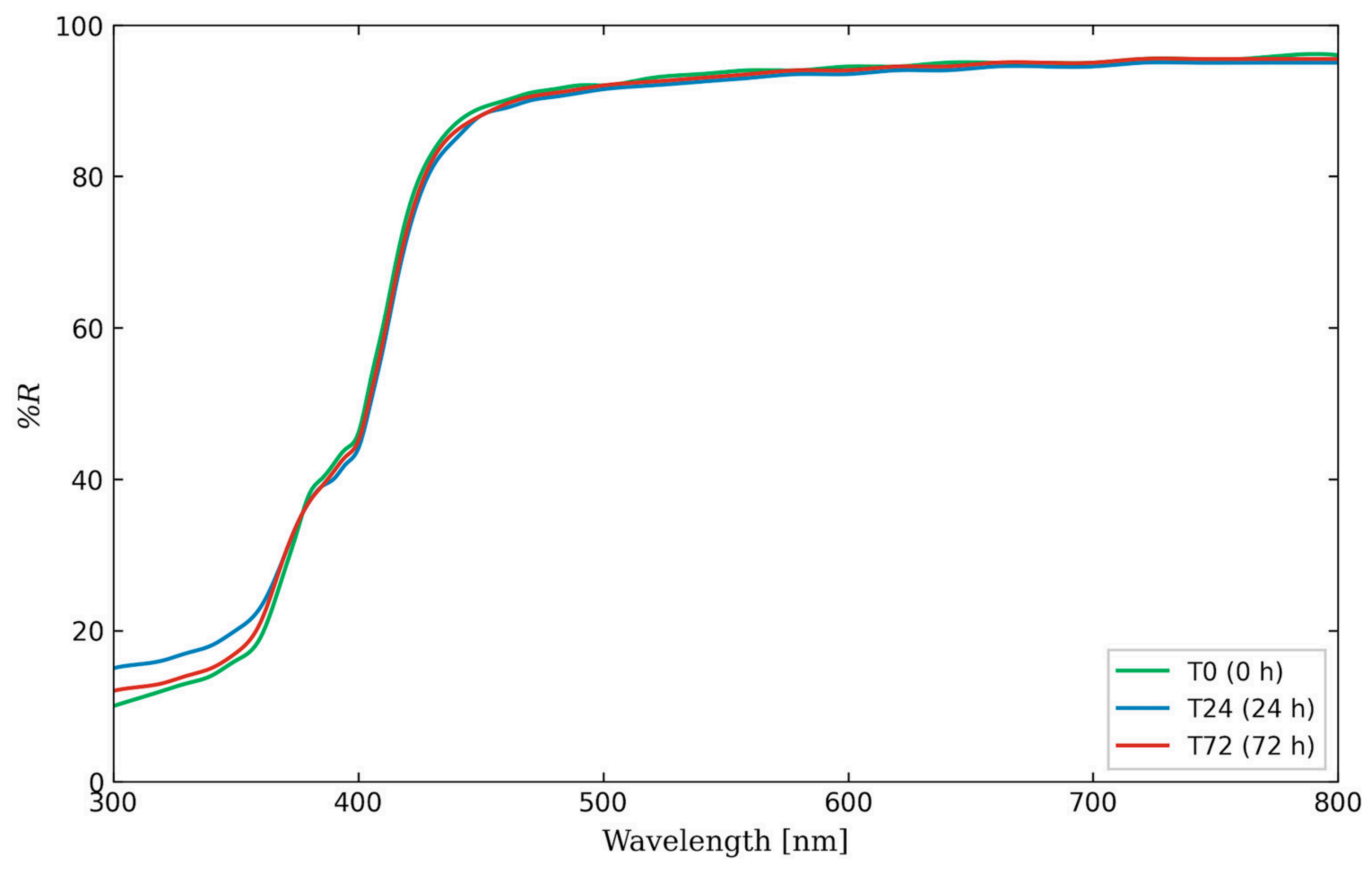
Diffuse reflectance spectra (%R) of Anycubic Bio Resin White specimens at baseline (T0, green), after 24 h immersion (T24, blue), and after 72 h immersion (T72, red) in artificial saliva at 37 °C. Wavelength range: 300–800 nm.

The spectral profile of Anycubic Bio Resin White (Figure 4) diverged markedly from the two dental-grade materials. A steep reflectance step between 400 and 420 nm separated a low-reflectance UV region (~10–15% at 300 nm) from an exceptionally high visible-range plateau approaching 95–96%. This sharp optical transition and the absence of a discernible absorption band at 350 nm suggest a simpler chromophore environment with no UV-blocking additives. The three time-point traces were virtually superimposable across the visible spectrum (>450 nm). A minor depression in the UV region (300–380 nm) was observed for T24 and T72 relative to T0, but this did not propagate into the visible domain (Figure 4).

## 4. Discussion

This study set out to isolate and compare the intrinsic, hydrolytically driven color change of three compositionally distinct printable resins, a skin-contact biocompatible acrylate, a urethane-dimethacrylate provisional medical device, and a high-UDMA nanocomposite, following static immersion in protein-free artificial saliva at 37 °C, deliberately excluding dietary chromogens and thermocycling so that the salivary contribution could be examined in isolation. Materials spanning different monomer, filler and photoinitiator chemistries were chosen precisely to test whether formulation, rather than immersion time, governs chromatic behaviour. Because the resulting subgroups were small (*n* = 4), the analysis combined parametric models (two-way and one-way ANOVA with effect sizes) with a pre-planned non-parametric cross-check (Kruskal–Wallis), and the interpretation below integrates both the statistical outcomes and their comparison with the recent literature. The results partially support the rejection of the null hypothesis: although the one-way ANOVA on ΔE did not reach conventional significance at either time point, the non-parametric Kruskal–Wallis test was significant at T72 (*p* = 0.024), and the consistently large effect sizes (Cohen’s d > 1.6 for Anycubic versus GC) indicate clinically meaningful differences that the study was underpowered to detect parametrically.

### 4.1. Material Composition as the Primary Determinant of Chromatic Behavior

The two-way ANOVA demonstrated that material identity was by far the dominant factor influencing all CIELAB parameters, with partial eta-squared values ranging from 0.774 (L*) to 0.964 (a*). This finding was expected given that the three resins differ fundamentally in monomer composition, filler content, and photoinitiator chemistry. The near-total explained variance for a* (η^2^p = 0.964) reflects that the chromatic axis positions are largely intrinsic properties of the material determined during formulation and remain stable across immersion times. The absence of a significant main effect of Time on any CIELAB parameter is notable but should be interpreted with caution [48]. The effect size for b* (η^2^p = 0.165) was in the large range, and the *p*-value (0.088) approached significance, suggesting that changes along the yellow-blue axis were occurring but that the study lacked sufficient power to confirm them statistically. A post hoc power analysis using the observed effect size and *n* = 4 per cell yields an estimated power of approximately 0.35 for detecting this effect at α = 0.05, which is substantially below the conventional threshold of 0.80 [10]. These observations are consistent with recent reports in which material formulation, printing orientation and post-curing regimen, rather than the aging medium alone, emerged as the principal drivers of the optical behaviour of additively manufactured resins [41,42]. The predominance of a b*-directed shift observed here also parallels findings that aging and surface challenges act preferentially on the yellow–blue coordinate of 3D-printed provisional materials [33,43].

### 4.2. Differential Chromatic Stability Among Materials

Anycubic Bio Resin White exhibited the highest chromatic stability, with mean ΔE values of 0.90 at T24 and 0.66 at T72, both well below the perceptibility threshold. The relatively high viscosity of this resin (470–520 mPa·s) suggests a formulation with higher molecular weight oligomers and potentially greater crosslink density after curing, both of which would limit water diffusion into the polymer network [4,10]. Additionally, the absence of inorganic fillers eliminates the potential for filler-matrix debonding and associated light-scattering changes that can occur in filled composite systems. The relatively simple unfilled acrylate matrix may also contribute to a more uniform post-cured network with fewer light-scattering interfaces. It should be noted that this observation is limited to a 72 h immersion window; longer exposures may reveal different degradation kinetics. GC Temp Print showed the highest mean ΔE at T24 (4.48 ± 3.07), with 75% of specimens exceeding the clinical acceptability threshold. The high baseline variability among GC control specimens (T0 L* range: 90.24 to 94.48, SD = 1.96) suggests that inter-specimen inconsistency in the printing or post-curing process may have contributed to the apparent color shift. When the reference value (mean T0) is derived from a heterogeneous baseline group, individual specimens at T24 may appear to have shifted substantially even if the actual immersion-induced change was modest. The high UDMA content (50–75%) in GC Temp Print is generally associated with dense crosslinked networks and limited water uptake [11,27]; thus, the elevated ΔE values may reflect measurement artifacts related to baseline heterogeneity rather than true hydrolytic degradation. VOCO V-Print c&b temp exhibited intermediate ΔE values (2.25 at T24, 3.79 at T72) with a progressive increase over time, consistent with a diffusion-driven process. The directional shift was primarily on the b* axis (from 15.52 at T0 to 12.99 at T72), indicating a reduction in yellowness. This pattern is consistent with the release of residual photoinitiator or co-initiator species, which often contribute a slight yellow tint to the uncured or partially cured resin [8,9]. The aliphatic urethane dimethacrylate backbone of V-Print was designed to resist yellowing, but the observed b* decline may indicate effective removal of chromogenic residues rather than matrix degradation [6]. The comparatively large ΔE of the nanocomposite and, to a lesser extent, of the urethane-dimethacrylate resin is in line with studies reporting that filled and dental-grade printable resins are more susceptible than simpler unfilled matrices to immersion- and aging-induced color change, an effect frequently attributed to filler–matrix interfaces and residual photoinitiator chemistry [33,42,43]. Where the present magnitudes exceed those reported elsewhere, the discrepancy is most plausibly explained by differences in resin composition, post-curing protocol, aging medium and the between-subjects baseline used to compute ΔE [41].

### 4.3. The Discrepancy Between Parametric and Non-Parametric Results

An important finding was the divergence between the parametric and non-parametric analyses at T72. The one-way ANOVA was not significant (*p* = 0.070), while the Kruskal–Wallis test was (*p* = 0.024). This discrepancy is explained by the distributional properties of the data: at T72, the four Anycubic specimens were tightly clustered at the low end of the ΔE distribution (range: 0.11 to 1.14), whereas VOCO and GC specimens were spread over a wider range (1.55 to 7.42). In such configurations, rank-based methods are more efficient because they are not inflated by the large within-group variance that depresses the F-statistic in ANOVA. This pattern illustrates the value of complementary parametric and non-parametric approaches when working with small samples. The larger partial eta-squared for the b* Material × Time interaction, although not statistically significant, is consistent with this pattern and suggests that any incipient time-dependent change is concentrated on the yellow–blue axis; given the limited power at *n* = 4, however, this remains a hypothesis to be tested in adequately powered designs rather than an established effect. The wide confidence intervals, several of which include zero, are consistent with the limited precision expected at *n* = 4 per group and reinforce the exploratory interpretation of these estimates.

### 4.4. Clinical Implications

From a clinical perspective, the threshold analysis provides the most directly actionable information. Anycubic Bio Resin White maintained color stability within clinically imperceptible limits throughout the 72 h immersion period, suggesting adequate short-term performance in environments limited to salivary exposure without additional chromogenic agents. It should be emphasized, however, that Anycubic Bio Resin White is a skin-contact biocompatible resin that is neither intended nor approved for intraoral use; its favourable in vitro colour stability therefore does not indicate suitability for intraoral clinical application. The finding that 75% of GC Temp Print specimens exceeded the acceptability threshold after only 24 h of immersion is clinically concerning for a material marketed for provisional intraoral use. However, as discussed above, this result must be contextualized against the high baseline variability of this material. Future studies employing larger sample sizes and within-subject designs (measuring the same specimen before and after immersion) would be needed to determine whether this reflects true clinical instability or a methodological limitation of the between-subjects design. VOCO V-Print c&b temp showed a progressive increase in ΔE that approached the acceptability threshold at 72 h (mean ΔE = 3.79). While the mean value was close to the 3.7 cutoff, only one of four specimens actually exceeded it. For short-duration provisional restorations (days to weeks), this material appears to offer acceptable chromatic performance under salivary conditions alone, though additional stressors such as dietary pigments or thermocycling would likely accelerate discoloration [5,38,40]. Beyond salivary exposure, dental materials in the oral cavity may also encounter acidic or alkaline substances from dietary or hygiene products, which have been shown to produce distinct patterns of structural and compositional alteration even in natural dental hard tissues. The interaction between such corrosive agents and 3D-printed resin surfaces warrants further investigation [12,49]. Clinically, these results imply that the choice of printable resin and strict control of the print-and-cure workflow matter more for short-term esthetic outcomes than the duration of salivary exposure per se. They also delimit the scope of the present conclusions: in the mouth, restorations are simultaneously exposed to salivary enzymes and proteins, biofilm and chromogenic bacteria, dietary pigments, thermal cycling, and mechanical brushing, all of which would be expected to amplify the modest changes observed here [7,14,18,43].

### 4.5. Limitations

Several limitations should be acknowledged. First, the small sample size (*n* = 4 per subgroup) limited statistical power; therefore, this study should be considered exploratory, and the observed effect sizes should guide future sample-size calculations. Second, the between-subjects design introduced inter-specimen variability, whereas a repeated-measures approach would provide greater sensitivity. Third, static immersion in artificial saliva without thermocycling or dietary chromogens was intentionally chosen to isolate intrinsic hydrolytic colour changes, although this limits clinical relevance. Fourth, only the CIE76 formula was used; future studies should also apply CIEDE2000, which better reflects perceived colour differences. Finally, the 72 h immersion period and qualitative spectral analysis from a single representative specimen limit the generalizability of the findings. Future research should include larger repeated-measures samples, longer aging periods, thermocycling, dietary staining, and complementary quantitative spectral and morphological analyses (e.g., SEM).

## 5. Conclusions

Material composition, rather than immersion duration, was the dominant determinant of the chromatic behaviour of the three printable resins, and immersion in artificial saliva for up to 72 h produced no statistically significant change in the individual CIELAB parameters.

In terms of pure chromatic stability, Anycubic Bio Resin White showed the smallest colour change, remaining below the acceptability threshold at all time points and below the perceptibility threshold at T72, with only a single specimen exceeding perceptibility at T24; it is, however, a skin-contact biocompatible material that is not intended for intraoral use, and its high lightness and low chroma limit its ability to reproduce natural dental esthetics. Among the dental-grade resins, V-Print c&b temp exhibited the most predictable chromatic behaviour under the specific short-term experimental conditions, whereas GC Temp Print showed the largest and most variable colour change, reflecting a marked sensitivity to printing and post-curing conditions. Because the present assessment was limited to short-term colour stability in artificial saliva, no conclusion regarding the overall clinical suitability of any material for intraoral provisional restorations can be drawn from these data alone.

Overall, resin selection and strict adherence to manufacturer-specified post-processing are decisive for the chromatic longevity of 3D-printed provisional restorations, and these short-term findings warrant confirmation in adequately powered studies incorporating longer exposure, thermocycling and dietary chromogens.

## Figures and Tables

**Table 1:** Composition and properties of the materials tested.

Material	Manufacturer	Intended Use	Primary Monomer (wt%)	Reactive Diluent (wt%)	Photoinitiator	Viscosity (mPa·s)	Other Physical Properties
Anycubic Bio Resin White	Anycubic Technology Co., Shenzhen, China	Skin-contact biocompatible resin, repurposed for provisional restorations	Acrylate oligomers *	Reactive monomers *	Not disclosed	470–520	Density 1.13–1.15 g/cm^3^; Shore D 83–85
V-Print c&b temp	VOCO GmbH, Cuxhaven, Germany	Class IIa medical device; long-term provisional restorations	Aliphatic UDMA (10–25%)	TEGDMA (2.5–5%)	Diphenyl(2,4,6-trimethylbenzoyl)phosphine oxide (1–2.5%)	Fluid (*n*/r)	*n*/r
GC Temp Print (Light)	GC Corporation, Tokyo, Japan	Nanocomposite printable provisional/temporary restorations	UDMA (50–75%)	Dimethacrylate (10–25%)	Phosphine oxide	2000	*n*/r

* Exact composition and weight fractions not disclosed by the manufacturer [44].

**Table 2:** Composition of the Fusayama–Meyer artificial saliva used as the immersion medium.

Component	Concentration (g/L)
NaCl	0.4
KCl	0.9
Urea	1.0
NaH_2_PO_4_	0.69
CaCl_2_·2H_2_O	0.795
Solution pH	5.2

**Table 3:** Descriptive statistics (mean ± SD) for CIELAB parameters and ΔE by material and immersion time (*n* = 4 per cell).

Material	Time	L* (Mean ± SD)	a* (Mean ± SD)	b* (Mean ± SD)	ΔE (Mean ± SD)	ΔE > 3.7
Anycubic	T0	94.32 ± 0.42	−1.85 ± 0.42	6.12 ± 0.41	—	—
	T24	93.83 ± 0.32	−1.95 ± 0.22	6.82 ± 0.50	0.90 ± 0.54	0/4
	T72	94.10 ± 0.24	−1.96 ± 0.32	6.52 ± 0.60	0.66 ± 0.49	0/4
VOCO	T0	88.49 ± 0.92	2.14 ± 0.29	15.52 ± 1.88	—	—
	T24	88.90 ± 1.43	2.32 ± 0.55	14.29 ± 2.51	2.25 ± 2.04	1/4
	T72	89.92 ± 1.74	1.98 ± 0.55	12.99 ± 3.26	3.79 ± 2.49	1/4
GC	T0	91.67 ± 1.96	0.47 ± 0.21	9.60 ± 2.25	—	—
	T24	93.91 ± 1.89	0.36 ± 0.24	5.76 ± 2.48	4.48 ± 3.07	3/4
	T72	93.17 ± 1.45	0.32 ± 0.35	6.52 ± 1.67	3.58 ± 1.91	2/4

**Table 4:** Two-way between-subjects ANOVA results for L*, a*, and b*.

Source	df	F	*p*	η^2^p	Effect	MSE
L*						
Material	2	46.237	<0.001	0.774	Large	
Time	2	1.563	0.228	0.104	Medium	
Material × Time	4	1.379	0.267	0.170	Large	
Error	27					1.760
a*						
Material	2	365.796	<0.001	0.964	Large	
Time	2	0.519	0.601	0.037	Small	
Material × Time	4	0.314	0.866	0.044	Small	
Error	27					0.137
b*						
Material	2	55.905	<0.001	0.805	Large	
Time	2	2.664	0.088	0.165	Large	
Material × Time	4	1.658	0.189	0.197	Large	
Error	27					3.923

**Table 5:** Proportion of specimens exceeding clinical color-change thresholds.

Material	Time	ΔE > 1.2 (*n*/4)	ΔE > 1.2 (%)	ΔE > 3.7 (*n*/4)	ΔE > 3.7 (%)
Anycubic	T24	1	25%	0	0%
Anycubic	T72	0	0%	0	0%
VOCO	T24	3	75%	1	25%
VOCO	T72	4	100%	1	25%
GC Temp Print	T24	3	75%	3	75%
GC Temp Print	T72	4	100%	2	50%

## Data Availability

The original contributions presented in the study are included in the article; further inquiries can be directed to the corresponding authors.
